# The Leukodystrophies HBSL and LBSL—Correlates and Distinctions

**DOI:** 10.3389/fncel.2020.626610

**Published:** 2021-01-26

**Authors:** Annapoorani Muthiah, Gary D. Housley, Matthias Klugmann, Dominik Fröhlich

**Affiliations:** Translational Neuroscience Facility and Department of Physiology, School of Medical Sciences, UNSW Sydney, Kensington, NSW, Australia

**Keywords:** leukodystrophy, leukoencephalopathy, aminoacyl-tRNA synthetases, aspartyl-tRNA synthetase, DARS1, DARS2, hypomyelination with brainstem and spinal cord involvement and leg spasticity, leukoencephalopathy with brainstem and spinal cord involvement and lactate elevation

## Abstract

Aminoacyl-tRNA synthetases (ARSs) accurately charge tRNAs with their respective amino acids. As such, they are vital for the initiation of cytosolic and mitochondrial protein translation. These enzymes have become increasingly scrutinized in recent years for their role in neurodegenerative disorders caused by the mutations of ARS-encoding genes. This review focuses on two such genes—*DARS1* and *DARS2*—which encode cytosolic and mitochondrial aspartyl-tRNA synthetases, and the clinical conditions associated with mutations of these genes. We also describe attempts made at modeling these conditions in mice, which have both yielded important mechanistic insights. *L*eukoencephalopathy with *b*rainstem and *s*pinal cord involvement and *l*actate elevation (LBSL) is a disease caused by a range of mutations in the *DARS2* gene, initially identified in 2003. Ten years later, *h*ypomyelination with *b*rainstem and *s*pinal cord involvement and *l*eg spasticity (HBSL), caused by mutations of cytosolic *DARS1*, was discovered. Multiple parallels have been drawn between the two conditions. The Magnetic Resonance Imaging (MRI) patterns are strikingly similar, but still set these two conditions apart from other leukodystrophies. Clinically, both conditions are characterized by lower limb spasticity, often associated with other pyramidal signs. However, perhaps due to earlier detection, a wider range of symptoms, including peripheral neuropathy, as well as visual and hearing changes have been described in LBSL patients. Both HBSL and LBSL are spectrum disorders lacking genotype to phenotype correlation. While the fatal phenotype of *Dars1* or *Dars2* single gene deletion mouse mutants revealed that the two enzymes lack functional redundancy, further pursuit of disease modeling are required to shed light onto the underlying disease mechanism, and enable examination of experimental treatments, including gene therapies.

## Introduction

Hypomyelination with Brainstem and Spinal Cord Involvement and Leg Spasticity (HBSL) and Leukoencephalopathy with Brainstem and Spinal Cord Involvement and Lactate Elevation (LBSL) both belong to a group of white matter disorders termed leukodystrophies. These conditions are caused by mutations of the *DARS1* and *DARS2* genes, respectively—two genes that encode aspartyl-tRNA synthetase enzymes responsible for the accurate charging of aspartate-specific transfer ribonucleic acid (tRNA) with aspartate (Scheper et al., 2007; Taft et al., 2013). Both enzymes are part of a larger group of enzymes known as aminoacyl-tRNA synthetases (ARSs), which are essential for protein translation. In humans, 36 ARSs exist without functional redundancy between them (Antonellis and Green, 2008).

ARSs can be divided into two subgroups based on their location of action—cytosolic and mitochondrial ARSs. In general, each ARS functions exclusively in one compartment and is responsible for the pairing of one of the 20 amino acids with its respective tRNA. Exceptions include glycyl-tRNA synthetase (GlyRS) and lysyl-tRNA synthetase (LysRS), which are capable of functioning in both compartments, and glutamyl-prolyl-tRNA synthetase (EPRS) which is able to pair both proline and glutamic acid with their respective tRNAs in the cytosolic compartment (Antonellis and Green, 2008). Interestingly, glutaminyl-tRNA synthetase (GlnRS) does not have a mitochondrial counterpart. Charging of mitochondrial tRNA^Gln^ to glutamine takes place through an indirect pathway, where “mischarging” with glutamic acid by mitochondrial glutamyl-tRNA synthetase (GluRS) is the first step (Nagao et al., 2009). While *DARS1* encodes the cytosolic aspartyl-tRNA synthetase (AspRS), *DARS2* encodes its mitochondrial counterpart (mt-AspRS). AspRS catalyzes the aminoacylation reaction exclusively in the cytosol, whereas mt-AspRS is transported from the cytosol to the mitochondrial matrix where it is involved in mitochondrial protein synthesis.

## Clinical Features

LBSL was first described in 2003, based on distinctive clinical features and a characteristic Magnetic Resonance Imaging (MRI) pattern (Van Der Knaap et al., 2003). Following this initial MRI-based diagnosis, a genetic basis for the condition was established in 2007, concluding mutations harbored in the nuclear-encoded *DARS2* gene to be the cause (Scheper et al., 2007). This was the first mitochondrial aminoacyl-tRNA synthetase gene to be identified as disease-causing in its mutated state and marked the beginning of many more conditions being linked to this group of enzymes. Subsequently, in 2013, HBSL was first characterized by Taft et al., who established mutations in the cytosolic AspRS encoding gene *DARS1* as the genetic cause for the condition (Taft et al., 2013). Both diseases are inherited in an autosomal recessive manner (Scheper et al., 2007; Taft et al., 2013). To date, more than 100 LBSL patients have been reported in the literature, while only 16 HBSL patients have been described in the past 7 years. However, the actual number of diagnosed HBSL patients is recognized to be considerably higher than the number of reported cases (pers. comm.).

Of note, neither condition features extra-neurological signs, particularly cardiac and skin symptoms, even though these tissues have high energy demand and cell turnover, respectively. This selective neurological phenotype is not unique to HBSL or LBSL but has been reported for many ARS-related diseases (Fuchs et al., 2019). The reason for the neurological preponderance of both HBSL and LBSL is the subject of ongoing studies. It is worth mentioning though that few ARS-related conditions do involve non-neural tissues; for example, some *AARS2* mutations are known to cause severe cardiomyopathy (Götz et al., 2011; Taylor et al., 2014), while other *AARS2* mutations do not.

The age at which symptoms are first detected is highly variable even within each condition. The onset of HBSL ranges from 4 months (Taft et al., 2013) to 18 years (Wolf et al., 2015). The average age of onset is 3.5 years; however, most patients experience first symptoms before the age of 1 year (56% of reviewed patients). For LBSL, the onset of disease occurs on average around the age of seven, but has been detected from birth (Steenweg et al., 2012) to as old as 43 years (Ikazabo et al., 2020). It is generally understood that, for both HBSL and LBSL, the earlier the onset of symptoms the more severe and rapid the progression of disease. For instance, one study found that LBSL patients who experience symptoms before the age of 1.5 years were more likely to be wheelchair-dependent than those experiencing symptoms after this age (Van Berge et al., 2014). At present, only seven LBSL patients out of 99 patients reviewed are known to be wheelchair-dependent (Serkov et al., 2004; Távora et al., 2007; Tylki-Szymanska et al., 2014; Shimojima et al., 2017; Yelam et al., 2019). These patients had onset of symptoms in infancy or early childhood. In terms of unsupported mobility, it can be argued that patients with HBSL experience more debilitating symptoms compared to patients with LBSL. All initially described HBSL patients with infantile-onset (<2 years old) were unable to mobilize independently (Taft et al., 2013). This is due to severe spasticity, a defining feature of HBSL, with lower limbs affected more severely than upper limbs. HBSL patients with childhood-onset presented with delayed motor development but were subsequently able to mobilize independently, albeit with repeated falls (Ong et al., 2020). HBSL patients with adult-onset have been shown to experience fewer and milder symptoms compared to infantile-onset patients (Wolf et al., 2015).

The age of onset also seems to predict symptomology. While there are some common clinical features that all LBSL and HBSL patients share, clinical presentation and progression seem to differ between different age groups. Infantile-onset LBSL patients typically present with hypotonia, areflexia and developmental delay or regression of achieved developmental milestones (Miyake et al., 2011; Steenweg et al., 2012; Grechanina and Zdubskaya, 2014; Tylki-Szymanska et al., 2014; Kohler et al., 2015; Navarro Vázquez et al., 2016; Yahia et al., 2018). Progression of the disease is also more rapid and typically followed by development of upper motor neuron (UMN) signs such as spasticity, hyperreflexia, and a positive Babinski sign. To date, eight deaths associated with LBSL have been reported and all of them belonged to this group of patients. Death usually occurred within 2 years of age, or ~1 year after the onset of symptoms with the exception of one patient who lived 7.5 years after onset of symptoms (Miyake et al., 2011; Orcesi et al., 2011; Steenweg et al., 2012; Navarro Vázquez et al., 2016; Rathore et al., 2017). The cause of death for these patients was either respiratory failure or respiratory tract infections. Children with disease onset in childhood or adolescence, on the other hand, typically present with gait difficulties, such as ataxia or unsteadiness and frequent falling. This is often accompanied by examination findings of spasticity, hyperreflexia, positive Babinski sign and distinct loss of proprioception and/or vibration sense. Ataxic gait in LBSL is also specified to be sensory in some reports (Labauge et al., 2007; Erturk et al., 2010; Synofzik et al., 2011; Tzoulis et al., 2012), further reinforced by findings of a positive Romberg sign (Linnankivi et al., 2004; Sharma et al., 2011; Martikainen et al., 2013; Ikazabo et al., 2020) and loss of proprioceptive and vibration sense in 11% and 49% of all clinically described LBSL cases, respectively (Table 1). In adult-onset patients, the presenting complaints also relate to difficulties in walking, however the clinical picture often includes more atypical findings. One patient, for example, experienced episodic attacks of ataxia (Synofzik et al., 2011), while another patient experienced predominantly unilateral (rather than bilateral) symptoms of spasticity and muscle weakness (Moore et al., 2012). These two patients incidentally also belonged to the few LBSL patients who did not possess the highly prevalent *DARS2* intron 2 splice site mutation.

**Table 1:** Overview of clinical characteristics reported in HBSL and LBSL.

**Clinical characteristics**	**HBSL (*n* = 16)^**a**^, % of cases reported**	**LBSL (*n* = 67)^**b**^, % of cases reported**
Male	56.3	46.3
Unsupported Walking	31.3	55.2
**Lower Limb Signs**		
Hypertonia	87.5	65.7
Hyperreflexia	87.5	52.2
Positive Babinski Sign	81.3	52.2
**Cerebellar Signs**		
Ataxia	50.0	70.1
Tremor	0.0	26.9
Dysmetria and/or Dysdiadochokinesia	18.8	11.9
Nystagmus	50.0	17.9
**Others**		
Dysarthria	18.8	19.4
Muscle Weakness	12.5	43.3
Muscle Cramps	0.0	4.5
Peripheral Neuropathy	0.0	28.4
Dorsal Column Dysfunction	6.3	49.3
Seizures	12.5	13.4
Cognitive Impairment	37.5	34.3
Visual Changes	62.5	11.9
Hearing Changes	0.0	4.5
Urinary Changes	6.3	9.0
Foot Deformities	0.0	14.9
Anemia	0.0	1.5

*Tremor encompasses intention tremor and reports of unspecified tremor. Visual changes include changes such as optic disc pallor, hypermetropia, optic atrophy, and myopia. Hearing changes include hyperacusis, hypoacusis, and sensorineural deafness. Urinary changes include increased urgency, incontinence, and changes in urine composition. Foot deformities include pes cavus, pes equinus, pes excavatus, hammertoe deformity, and contractures of the foot. Only data from full-text publications was included in this table. Characteristics were deemed to be not present if the feature was not mentioned, not investigated, or if they were stated to be normal (where applicable)*.

a *References: (Taft et al., 2013; Wolf et al., 2015; Ong et al., 2020)*.

b *References: (Van Der Knaap et al., 2003; Linnankivi et al., 2004; Serkov et al., 2004; Petzold et al., 2006; Labauge et al., 2007, 2011; Távora et al., 2007; Uluc et al., 2008; Erturk et al., 2010; Isohanni et al., 2010; Lin et al., 2010; Galluzzi et al., 2011; Mierzewska et al., 2011; Miyake et al., 2011; Orcesi et al., 2011; Sharma et al., 2011; Synofzik et al., 2011; Moore et al., 2012; Steenweg et al., 2012; Tzoulis et al., 2012; Cheng et al., 2013; Martikainen et al., 2013; Schicks et al., 2013; Alibas et al., 2014; Kassem et al., 2014; Tylki-Szymanska et al., 2014; Kohler et al., 2015; Navarro Vázquez et al., 2016; Lan et al., 2017; Shimojima et al., 2017; Cavusoglu et al., 2018; Gonzalez-Serrano et al., 2018; Werner et al., 2018; Yahia et al., 2018; Yelam et al., 2019; Ikazabo et al., 2020)*.

There are several notable exceptions to this generalized disease pattern in LBSL. The most significant is a male who experienced first symptoms at the age of 9 months (Kohler et al., 2015), where as an infant, he was irritable and had attention deficits. These worsened following a fever and respiratory infection, additionally resulting in poor eye contact and a delay in achieving motor milestones. Surprisingly, from 12.5 months of age, his condition improved to reach appropriate milestones for his age, including social smiling. His only residual symptom is mild ataxia that does not impede his ability to mobilize independently, including walking and running. It is also interesting that this patient is homozygous for *DARS2* mutations (c.172C>G; p.R58G). While the majority of LBSL patients are compound heterozygous, homozygosity has only been described in five LBSL patients from three unrelated families (Miyake et al., 2011; Synofzik et al., 2011; Kohler et al., 2015). Hence, homozygosity was initially presumed to be developmentally lethal until the first LBSL patient with homozygous *DARS2* mutations was described in 2011. Two of these patients (who are siblings) have experienced infantile-onset disease with severe disease course and subsequent death (Yamashita et al., 2013). Meanwhile, the third sibling experienced cognitive delay and severe motor deficits, and currently mobilizes by wheelchair.

Another patient with homozygous *DARS2* mutations experienced episodic bouts of ataxia that were induced by exercise, a highly unique disease presentation that has not been described in another LBSL patient (Synofzik et al., 2011). It was suggested that the finding of episodic ataxia might be incidental rather than the result of *DARS2* mutations (van der Knaap et al., 2014). Episodic ataxia, or recurring intermittent attacks of imbalance and incoordination, is a cardinal sign of a group of inherited diseases known as hereditary episodic ataxias (EA). Individuals with EA experience bouts of ataxia, of varying frequency and intensity, accompanied by other signs such as seizures and muscle fasciculation (Teive and Ashizawa, 2015). These attacks are often triggered by several environmental factors (Jen and Wan, 2018). The aforementioned patient, however, was not found to harbor any mutations for the most common EA types (Synofzik et al., 2011). Homozygous mutations, therefore, do not seem to be a reliable indicator of the prognosis in LBSL. In fact, genotype-phenotype correlations have been challenging to establish in this condition, and there are suggestions of additional genetic and non-genetic factors influencing disease presentation (Steenweg et al., 2012; Van Berge et al., 2014). This is further highlighted by the identification of a female who possessed the same two mutant *DARS2* alleles as her affected sister but experienced no clinical symptoms (Labauge et al., 2011).

Ultimately, the most common sign in LBSL is cerebellar ataxia and unsteady gait. Motor impairment is mainly caused by UMN dysfunction, however lower motor neuron (LMN) signs have also been described (Miyake et al., 2011; Steenweg et al., 2012; Grechanina and Zdubskaya, 2014; Yelam et al., 2019). Some other less frequent symptoms that have been observed in LBSL patients include unspecified tremor (Isohanni et al., 2010; Galluzzi et al., 2011; Sharma et al., 2011; Yamashita et al., 2013; Werner et al., 2018) and peripheral neuropathy (Linnankivi et al., 2004; Uluc et al., 2008), among others. Cognitive deficits are usually mild, if present, and typically affect information-processing speed, concentration, and short-term memory (Martikainen et al., 2013). An overview of reported LBSL and HBSL symptoms can be found in Table 1.

HBSL is less diverse in its clinical presentation, and there has only been one known death associated with HBSL thus far (pers. comm.). This patient experienced infantile onset of symptoms and lived to 9 years of age. The most common presentation in infants is regression in motor development or developmental delay (Taft et al., 2013). Some additional congenital abnormalities including spinal cord tethering (Taft et al., 2013), vertebral malformations, trigonocephaly and Chiari malformations (Ong et al., 2020) have also been noted in a few patients. In older-onset HBSL patients, spasticity is one of the first symptoms (Wolf et al., 2015). Most reported patients have experienced leg spasticity—to varying degrees—that have impaired their ability to walk. Gait has been described as diplegic (Taft et al., 2013) or in-toeing (Ong et al., 2020) in those able to mobilize. In more severe cases, upper limb involvement is also noted. HBSL is predominantly a UMN disorder, with consistent hypertonia, hyperreflexia and positive Babinski sign noted in nearly all patients (Table 1). Nystagmus has also been a common finding. To a lesser extent, axial hypotonia (Taft et al., 2013; Wolf et al., 2015), epilepsy (Taft et al., 2013), cognitive deficits (Taft et al., 2013; Ong et al., 2020), and headaches (Ong et al., 2020) have been noted in some patients. In two adult-onset patients, symptoms of dorsal column dysfunction and urinary changes have been identified, as seen in LBSL (Wolf et al., 2015). The main differences in the clinical picture of HBSL and LBSL, are the clear lack of peripheral neuropathy and LMN signs in HBSL patients, while significant leg spasticity is the predominant feature. No hearing changes have been noted in HBSL patients. Evidence of intrafamilial variations and weak genotype-phenotype correlations, are present in HBSL as well. This is supported by a report of a family with three affected siblings carrying the same mutations but experiencing different disease courses (Ong et al., 2020).

In both conditions, a number of patients experience symptoms following clear precipitating events—for instance, fever, viral illness, or head trauma—either resulting in the first onset of symptoms (Wolf et al., 2015; Yahia et al., 2018) or further neurological deterioration (Uluc et al., 2008; Taft et al., 2013). Most patients experiencing such an exacerbation of symptoms usually recover to a new baseline with some residual deficits (Serkov et al., 2004; Isohanni et al., 2010; Wolf et al., 2015).

## Neuroimaging and Diagnosis

MRI is the tool of choice when investigating white matter (WM) diseases. Most leukodystrophies can be accurately diagnosed with this modality, as they present with a unique MRI signature (Schiffmann and van der Knaap, 2009). However, between HBSL and LBSL, MRI is not highly discriminatory, as both conditions affect similar brain structures and tracts (Taft et al., 2013; Wolf et al., 2015). While MRI remains the main imaging modality to initially identify HBSL and LBSL patients, genetic analysis is necessary to subsequently differentiate between the two conditions and is currently the only means to a definite diagnosis.

Nonetheless, prior to genetic analysis, a preliminary LBSL diagnosis can be made on an MRI basis. With the revised criteria proposed by Steenweg et al., three major criteria must be fulfilled—signal changes in the cerebral WM, involvement of the dorsal columns and lateral corticospinal tracts in the spinal cord and the involvement of either the pyramidal or medial lemniscal tracts in the medulla (Steenweg et al., 2012). These three major criteria are largely satisfied in all genetically confirmed LBSL patients (Table 2). Abnormal cerebral WM is typically observed in the periventricular region, with distinct sparing of u-fibers. A sizeable number of patients have also displayed WM changes extending into the deep cerebral WM (Kassem et al., 2014). Changes are usually bilateral and inhomogeneous, and sometimes described as spotty. Cerebral WM has also been noted to have bilateral focal lesions in some patients (Erturk et al., 2010). Rarely, additional involvement of the u-fibers has been reported (Galluzzi et al., 2011; Orcesi et al., 2011, Yamashita et al., 2013).

**Table 2:** Overview of MRI changes reported in LBSL.

**MRI Changes**	**LBSL (*n* = 83)^**a**^, proportion of patients (%)**
**Supratentorial**	
Cerebral WM with subcortical sparing^*^	92.8
Posterior part of the corpus callosum	77.1
Posterior limb of the internal capsule	81.9
**Infratentorial**	
Lateral corticospinal tracts or dorsal columns of the spinal cord	89.2
Pyramidal tract and/or medial lemniscus of the brainstem	94.0
Superior cerebellar peduncles	73.5
Inferior cerebellar peduncles	69.9
Intraparenchymal trajectories of the trigeminal nerve	63.9
Mesencephalic trigeminal tracts	47.0
Anterior spinocerebellar tracts of the medulla oblongata	32.5
Cerebellar white matter	88.0

*Adapted, with some modifications, from the MRI criteria described by Steenweg et al. (2012). Only data from full-text publications was included in this table. MRI changes were deemed to be not present if the feature was not mentioned, not investigated, or if they were stated to be normal*.

* *Patients with cerebral white matter changes, but without subcortical u-fiber sparing, were not included in this count*.

a *References: (Van Der Knaap et al., 2003; Linnankivi et al., 2004; Serkov et al., 2004; Petzold et al., 2006; Labauge et al., 2007, 2011; Távora et al., 2007; Uluc et al., 2008; Erturk et al., 2010; Isohanni et al., 2010; Lin et al., 2010; Galluzzi et al., 2011; Mierzewska et al., 2011; Miyake et al., 2011; Orcesi et al., 2011; Sharma et al., 2011; Synofzik et al., 2011; Moore et al., 2012; Steenweg et al., 2012; Tzoulis et al., 2012; Cheng et al., 2013; Martikainen et al., 2013; Schicks et al., 2013; Alibas et al., 2014; Kassem et al., 2014; Tylki-Szymanska et al., 2014; Kohler et al., 2015; Navarro Vázquez et al., 2016; Lan et al., 2017; Shimojima et al., 2017; Cavusoglu et al., 2018; Gonzalez-Serrano et al., 2018; Werner et al., 2018; Yahia et al., 2018; Yelam et al., 2019; Ikazabo et al., 2020)*.

In conjunction with the major criteria, at least one minor criterion should also be satisfied for diagnosis of LBSL. These minor criteria include signal changes in the cerebellar WM, posterior corpus callosum, posterior limb of the internal capsule (IC), superior and inferior cerebellar peduncles, intraparenchymal trajectories of the trigeminal nerve, mesencephalic tracts of the trigeminal nerve and anterior spinocerebellar tract in the medulla (Steenweg et al., 2012). A majority of patients display changes in the cerebellar WM, corpus callosum and posterior limb of the IC (Table 2). Involvement of the trigeminal nerve tracts and spinocerebellar tracts have been reported to a lesser extent (Table 2). Apart from these typical MRI changes, some atypical findings have been reported in genetically confirmed LBSL patients. These include the bilateral involvement of gray matter structures such as the globus pallidus (Galluzzi et al., 2011; Steenweg et al., 2012), red nucleus (Galluzzi et al., 2011), thalamus (Orcesi et al., 2011) and dentate nucleus (Galluzzi et al., 2011; Iyer and Philip, 2011; Orcesi et al., 2011; Yahia et al., 2018). Furthermore, cerebral (Tzoulis et al., 2012; Yamashita et al., 2013; Yahia et al., 2018) and cerebellar atrophy (Van Der Knaap et al., 2003; Yahia et al., 2018) has been reported, in addition to cerebellar hypoplasia (Isohanni et al., 2010). A singular case of calcifications of the cerebral WM and other regions typically affected by LBSL was identified by computed tomography (CT) scanning (Orcesi et al., 2011).

For HBSL, no such MRI criteria have been defined yet, however, some characteristic MRI features can be observed in the majority of patients (Table 3). These include homogenous cerebral WM changes (Taft et al., 2013; Wolf et al., 2015; Ong et al., 2020), abnormal posterior limb of the IC and hyperintensity of the corpus callosum on T2-weighted images (Taft et al., 2013; Wolf et al., 2015). Thinning of the corpus callosum has also been reported in some patients (Taft et al., 2013). In one HBSL patient, the additional involvement of the anterior corticospinal tract was observed on spinal MRI (Wolf et al., 2015). As seen in LBSL, the superior and inferior cerebellar peduncles, the pyramidal tracts in the medulla and spinal cord, and the dorsal columns of the spinal cord were affected in most patients. The cerebellar WM was also affected in several patients. Unlike in LBSL, medial lemniscal involvement was only observed in one patient (Taft et al., 2013), while no involvement of the trigeminal nerve tracts, spinocerebellar tracts and gray matter structures have been described for HBSL so far. The latter findings are also not common in LBSL and are therefore not reliable differentiators between the two conditions.

**Table 3:** MRI features reported in HBSL.

**MRI Changes**	**HBSL (*n* = 15)^**a**^, proportion of patients (%)**
**Supratentorial WM**	
Homogenous Cerebral WM Abnormality	80.0
Focal/Patchy Cerebral WM Changes	20.0
Hyperintense Corpus Callosum	60.0
Abnormal Posterior Limb of Internal Capsule	60.0
**Brainstem**	
Abnormal Signal of Pyramidal Tract	53.3
Abnormal Signal of Medial Lemniscus	6.7
**Cerebellum**	
Abnormal Signal of White Matter	26.7
Abnormal Signal of Superior Cerebellar Peduncle	60.0
Abnormal Signal of Inferior Cerebellar Peduncle	53.3
**Spinal Cord**	
Abnormal Signal of Dorsal Columns^*^	100.0
Abnormal Signal of Lateral Corticospinal Tracts^**^	90.9

*Only data from full-text publications were included in this table. MRI changes were deemed to be not present if the feature was not mentioned, or if they were stated to be normal*.

* *n = 13, as this feature was not investigated in two patients (Taft et al., 2013)*.

** *n = 11, as this feature was not investigated in four patients (Taft et al., 2013)*.

a *References: (Taft et al., 2013; Wolf et al., 2015; Ong et al., 2020)*.

As the name suggests, lactate elevation detected by magnetic resonance spectroscopy (MRS) is often thought to be a defining feature of LBSL. However, elevated lactate levels were only detected in 68% of LBSL patients. Furthermore, even in patients where lactate was elevated, this elevation fluctuated over time (Van Der Knaap et al., 2003; Isohanni et al., 2010). Therefore, while raised lactate levels may be a good indicator for LBSL, an absence of lactate elevation does not necessarily exclude it, or automatically confirm HBSL. It has been suggested that lactate elevation might be more common in early-onset LBSL (Werner et al., 2018). Additional MRS analyses of affected WM regions of LBSL patients also revealed decreased N-acetylaspartate (NAA), elevated choline (Cho), elevated myoinositol (mIns) (Van Der Knaap et al., 2003) and occasionally elevated creatine (Cr) (Erturk et al., 2010). While no lactate elevation has been described in HBSL patients, one case report of HBSL showed mildly increased Cho in one patient and decreased NAA in another patient (Ong et al., 2020). A decrease in NAA, as observed in 52% of LBSL patients reviewed, implicates axonal damage or loss (Moffett et al., 2007). Increased Cho levels suggest demyelination (Van Der Knaap et al., 2003; Galluzzi et al., 2011) while an increase in mIns is a sign for gliosis (Chang et al., 2013). Finally, Cr when elevated can imply disturbance in cell metabolism (Rackayova et al., 2017). Taken together, a more detailed analysis of the metabolic changes in the brains of LBSL and HBSL patients could shed some light on the underlying mechanisms of these conditions.

Diffusion weighted imaging (DWI) has also been performed in LBSL patients, revealing diffusion restriction in parts of the affected WM (Mierzewska et al., 2011; Alibas et al., 2014; Kassem et al., 2014). DWI indicated that there are progressive changes that seem to correlate with different stages of the disease (Steenweg et al., 2011). Initially, the observed diffusion restriction is attributed to myelin-splitting and oedema within myelin. This is followed by shifting of water to the interstitial space, resulting in high T2 signals (Steenweg et al., 2011). Thereafter, the loss of water in the interstitial space indicates the final component of the disease process, leading to intermediate T2 signals (Steenweg et al., 2011). These findings have been supported by post-mortem histopathological observations in two LBSL patients, which showed intra-lamellar splitting of myelin coupled with vacuolar, spongiform degeneration of the cerebral and cerebellar WM (Yamashita et al., 2013). Similar DWI studies in HBSL patients, in addition to more detailed MRS studies, will provide valuable insight into the progression of this leukodystrophy.

Although MRI is a reliable tool for diagnosis, clinical severity of LBSL does not always reflect the MRI results. Labauge et al. described a sibling to a proband who had characteristic MRI changes typically seen in affected LBSL patients, yet did not show any symptoms (Labauge et al., 2011). Genetic testing revealed that this person was a compound heterozygous carrier of *DARS2* mutations. Furthermore, there appears to be a delay between MRI changes and the onset of clinical symptoms in LBSL (Isohanni et al., 2010). These findings indicate that MRI could potentially be used to provide an early, pre-symptomatic diagnosis of the disease. An adult-onset LBSL patient was described to have solely right-sided spasticity, yet pyramidal tracts were equally affected bilaterally throughout their entire length (Moore et al., 2012), suggesting that MRI results do not always match clinical symptoms. MRI findings in HBSL patients are generally more consistent with the clinical presentation and MRI can be used to differentiate between infantile and adult-onset patients. Changes in adult-onset patients are more localized and less severe (Wolf et al., 2015). Moreover, in the least affected sibling of three reported HBSL patients, cerebral WM changes were noted to be patchy and less widespread compared to the more severely affected siblings (Ong et al., 2020).

Apart from HBSL and LBSL, there are other white matter disorders that affect similar regions of the CNS, which could potentially result in misdiagnosis. For example, one patient with mutations in the iron-sulfur cluster assembly 2 (ISCA2) gene was initially diagnosed with LBSL based on MRI and clinical features, prior to genetic testing (Toldo et al., 2018). Mutations in this gene cause multiple mitochondrial dysfunctions syndrome 4 (MMDS4), which has MRI features similar to LBSL and HBSL including signal changes in the periventricular WM, corpus callosum, posterior limb of the IC, cerebellar WM and cerebellar peduncles (Al-Hassnan et al., 2015; Alaimo et al., 2018; Toldo et al., 2018; Eidi and Garshasbi, 2019; Hartman et al., 2020). Additionally, a few MMDS4 patients also display lactate peaks on MRS (Alfadhel, 2019), as well as diffusion restriction in some affected WM regions on DWI (Alaimo et al., 2018; Toldo et al., 2018; Hartman et al., 2020). However, some MMDS4 patients show signal abnormality of the cerebral peduncles (Alaimo et al., 2018), as well as glycine elevation (Alfadhel, 2019), which have not been reported in LBSL or HBSL. Similarities have also been drawn between multiple sclerosis (MS) and HBSL and LBSL (Mierzewska et al., 2011; Wolf et al., 2015). This is likely because MS is seen to affect the same regions of the brain—the periventricular WM, brainstem, cerebellar peduncles, cerebellum and spinal cord (Filippi et al., 2019). Unlike MS however, abnormalities of WM in HBSL and LBSL are noted to be more symmetrical, and to affect specific tracts (Mierzewska et al., 2011; Filippi et al., 2019). Especially in the spinal cord, MS lesions are seen to be limited to a maximum of two vertebral levels and to be focal rather than affecting whole tracts (Wolf et al., 2015; Filippi et al., 2019). Adult-onset HBSL patients presenting with only focal MRI changes were originally misdiagnosed with MS, prior to genetic testing. As a result, treatment with steroids was initiated, resulting in transient recovery of symptoms (Wolf et al., 2015). The responsiveness to steroids paired with a relapsing-remitting course and focal white matter and spinal cord signal changes on MRI normally indicate inflammatory demyelinating diseases such as MS or neuromyelitis optica (Wolf et al., 2015). The similar presentation of adult-onset HBSL cases and MS patients indicates that the actual number of HBSL cases might be higher than originally anticipated and it has been suggested that HBSL be included in the differential diagnosis of CNS inflammatory diseases (Wolf et al., 2015).

As previously mentioned, the definite diagnosis of HBSL and LBSL requires genetic testing. The first LBSL cases reported were diagnosed using genome-wide linkage analysis to determine the disease-causing mutations (Scheper et al., 2007). The first diagnosis of HBSL was made using a novel technology at the time—full-genome trio sequencing (Taft et al., 2013). Using this method, the entire genome of the patient and both parents are sequenced and compared to limit the number of candidate genes. In a clinical setting, however, standard sequencing techniques such as next-generation sequencing (NGS) and Sanger sequencing are used to obtain a genetic diagnosis when HBSL and LBSL are already suspected based on clinical and MRI findings, which is faster and more cost-efficient than whole genome sequencing (WGS). Some LBSL patients with large deletion mutations have been reported (Lan et al., 2017). These mutations are not detected by standard sequencing techniques and it is recommended to follow up with copy number analysis to provide a diagnosis (Lan et al., 2017). Whole exon deletions have not been described for HBSL.

## Mechanism of Disease

With the use of WGS, mutations in the nuclear genes *DARS* and *DARS2* were identified to be the cause of HBSL and LBSL, respectively. *DARS1* is located on chromosome 2 in the 2q21.3 region and encodes AspRS (Taft et al., 2013). *DARS2* is located on chromosome 1 in the 1q25.1 region and encodes the mitochondrial counterpart mt-AspRS (Scheper et al., 2007). While AspRS acts exclusively in the cytosol, mt-AspRS is transported into the mitochondrial matrix and is responsible for the translation of mitochondrial transcripts.

The majority of LBSL and HBSL patients are compound heterozygous. Only very few LBSL patients are homozygous (5.6% of reported cases). In comparison, 37.5% of reported HBSL cases are homozygous. All *DARS1* mutations identified thus far are missense mutations, affecting amino acids primarily located in and around the catalytic domain of AspRS (Taft et al., 2013; Wolf et al., 2015; Ong et al., 2020) (Table 4). These mutations are thought to impair aminoacylation directly through changes of the active site, or through destabilization of the side chain interactions with the active site (Taft et al., 2013). Many of these mutations occur in highly conserved amino acids of AspRS (Taft et al., 2013; Ong et al., 2020).

**Table 4:** Summary of known HBSL mutations and their corresponding amino acid change.

**Nucleotide change**	**Amino acid change**	**Reference(s)**
c.536G>A	p.R179K	Ong et al., 2020
c.599C>G	p.S200C	Wolf et al., 2015
c.766A>C	p.M256L	Taft et al., 2013
c.821C>T	p.A274V	Taft et al., 2013
c.830C>T	p.S277F	Wolf et al., 2015
c.839A>T	p.H280L	Wolf et al., 2015
c.1099G>T	p.D367Y	Taft et al., 2013
c.1099G>C	p.D367H	Wolf et al., 2015
c.1277T>C	p.L426S	Wolf et al., 2015
c.1379G>A	p.R460H	Taft et al., 2013
c.1391C>T	p.P464L	Taft et al., 2013
c.1459C>T	p.R487C	Taft et al., 2013
c.1480C>G	p.R494G	Taft et al., 2013; Ong et al., 2020
c.1480C>T	p.R494C	Taft et al., 2013

In contrast, LBSL-causing mutations are scattered throughout the length of the *DARS2* gene (Table 5). These mutations include missense, non-sense, deletion, and splice site mutations. Most patients carry a mutation in the intron 2 splice acceptor region, several nucleotides upstream of exon 3 (Scheper et al., 2007). A mutation in this region affects about 88% of LBSL patients reviewed, and one mutation at this site (c.228-20_21delTTinsC) is estimated to have a carrier frequency of 1:95 in the Finnish population (Isohanni et al., 2010). A mutation at this splice acceptor region causes the exclusion of exon 3 in mt-AspRS transcripts. This exclusion ultimately results in a frame shift (p.R76SfsX5) and premature stop (van Berge et al., 2012), possibly producing a truncated, non-functional protein. It is important to note that this mutation is considered “leaky,” allowing low-level production of wild-type, functional mt-AspRS protein from transcripts including exon 3 (van Berge et al., 2012). Remarkably, this splicing defect is particularly profound within neural cells, and specifically in neurons, significantly lowering the amount of functional mt-AspRS produced by these cells (van Berge et al., 2012). Even in healthy individuals with two unaffected *DARS2* copies, splicing of this region is less efficient in neurons compared to other cell types. When this mutation is coupled with another *DARS2* mutation on the second allele, the amount of wild-type protein produced becomes insufficient (van Berge et al., 2012). The splicing differences between individuals potentially explain the large clinical variation among patients, including intrafamilial variations where affected family members possess the same *DARS2* mutations (Labauge et al., 2011). Despite its high prevalence, homozygous mutations at this site have only been reported in one family with three affected siblings (Miyake et al., 2011). Additionally, one patient carrying two different mutations at this site has been reported (Orcesi et al., 2011). In HBSL, the most prevalent mutation site is in exon 9 of the *DARS1* gene, causing a single amino acid change from methionine to leucine. This mutation was found in four patients from three different families (Taft et al., 2013).

**Table 5:** Summary of known LBSL mutations and their corresponding amino acid change.

**Nucleotide change**	**Amino acid change**	**Reference(s)**
c.133A>G	p.S45G	Scheper et al., 2007
c.172C>G	p.R58G	Kohler et al., 2015
c.228-10C>A	p.R76SfsX5	Scheper et al., 2007; Steenweg et al., 2012
c.228-11C>G	p.R76SfsX5	Scheper et al., 2007; Schicks et al., 2013
c.228-12C>A	p.R76SfsX5	Galluzzi et al., 2011; Sharma et al., 2011
c.228-12C>G	p.R76SfsX5	Orcesi et al., 2011; Steenweg et al., 2012
c.228-15C>A	p.R76SfsX5	Scheper et al., 2007; Steenweg et al., 2012; Yelam et al., 2019
c.228-15C>G	p.R76SfsX5	Scheper et al., 2007; Shimojima et al., 2017
c.228-16C>A	p.R76SfsX5	Scheper et al., 2007; Lin et al., 2010; Steenweg et al., 2012; Lan et al., 2017
c.228-16C>G	p.R76SfsX5	Labauge et al., 2011; Steenweg et al., 2012
c.228-20_21delTTinsC	p.R76SfsX5	Scheper et al., 2007; Uluc et al., 2008; Erturk et al., 2010; Isohanni et al., 2010; Mierzewska et al., 2011; Tzoulis et al., 2012; Cheng et al., 2013; Martikainen et al., 2013; Alibas et al., 2014; Tylki-Szymanska et al., 2014; Cavusoglu et al., 2018; Gonzalez-Serrano et al., 2018; Werner et al., 2018
c.228-22T>A	p.R76SfsX5	Miyake et al., 2011
c.228-24insT	p.R76SfsX5	Orcesi et al., 2011
c.259G>A	p.D87N	Rathore et al., 2017
c.295-2A>G	p.A100_P132del	Scheper et al., 2007
c.396+2T>G	p.A100_P132del	Scheper et al., 2007
c.358_359delinsTC	p.G120S	Shimojima et al., 2017
c.374G>A	p.R125H	Ikazabo et al., 2020
c.397-2A>G	p.M134_K165del	Scheper et al., 2007; Erturk et al., 2010
c.492+2T>C	p.M134_K165del	Scheper et al., 2007; Isohanni et al., 2010; Galluzzi et al., 2011; Moore et al., 2012; Steenweg et al., 2012; Martikainen et al., 2013; Alibas et al., 2014; Grechanina and Zdubskaya, 2014; Tylki-Szymanska et al., 2014
c.416T>C	p.I139T	Steenweg et al., 2012
c.455G>T	p.C152F	Scheper et al., 2007; Isohanni et al., 2010; Steenweg et al., 2012; Cavusoglu et al., 2018; Werner et al., 2018
c.473A>T	p.E158V	Moore et al., 2012
c.536G>A	p.R179H	Scheper et al., 2007
c.550C>A	p.Q184K	Scheper et al., 2007
c.563G>A	p.R188Q	Yahia et al., 2018
c.617_663del	p.F207CfsX25	Schicks et al., 2013
c.716T>C	p.L239P	Lin et al., 2010
c.742C>A	p.Q248K	Scheper et al., 2007
c.745C>A	p.L249I	Labauge et al., 2011
c.749T>C	p.L250P	Steenweg et al., 2012
c.787C>T	p.R263X	Scheper et al., 2007
c.788G>A	p.R263Q	Scheper et al., 2007; Rathore et al., 2017
c.822_825del	p.R274SfsX9	Steenweg et al., 2012
c.850G>A	p.E284K	Cheng et al., 2013
c.1013G>A	p.G338E	Gonzalez-Serrano et al., 2018; Ikazabo et al., 2020
c.1069C>T	p.Q357X	Sharma et al., 2011
c.1129_1191	p.K377_Q397del	Lan et al., 2017
c.1272_1273G>C	p.E424NfsX1	Scheper et al., 2007
c.1273G	p.E425X	Scheper et al., 2007
c.1345-17del13	p.C449_K521del	Uluc et al., 2008
c.1395_1396delAA	p.G467SfsX7	Tzoulis et al., 2012
c.1564_1674del	p.A522_K558del	Scheper et al., 2007
c.1679A>T	p.D560V	Scheper et al., 2007
c.1762C>G	p.L588V	Yahia et al., 2018
c.1825C>T	p.R609W	Synofzik et al., 2011
c.1837C>T	p.L613F	Scheper et al., 2007
c.1875C>G	p.L626V	Scheper et al., 2007
c.1876T>A	p.L626Q	Scheper et al., 2007
c.1886A>G	p.Y629C	Scheper et al., 2007

**Figure 1:**
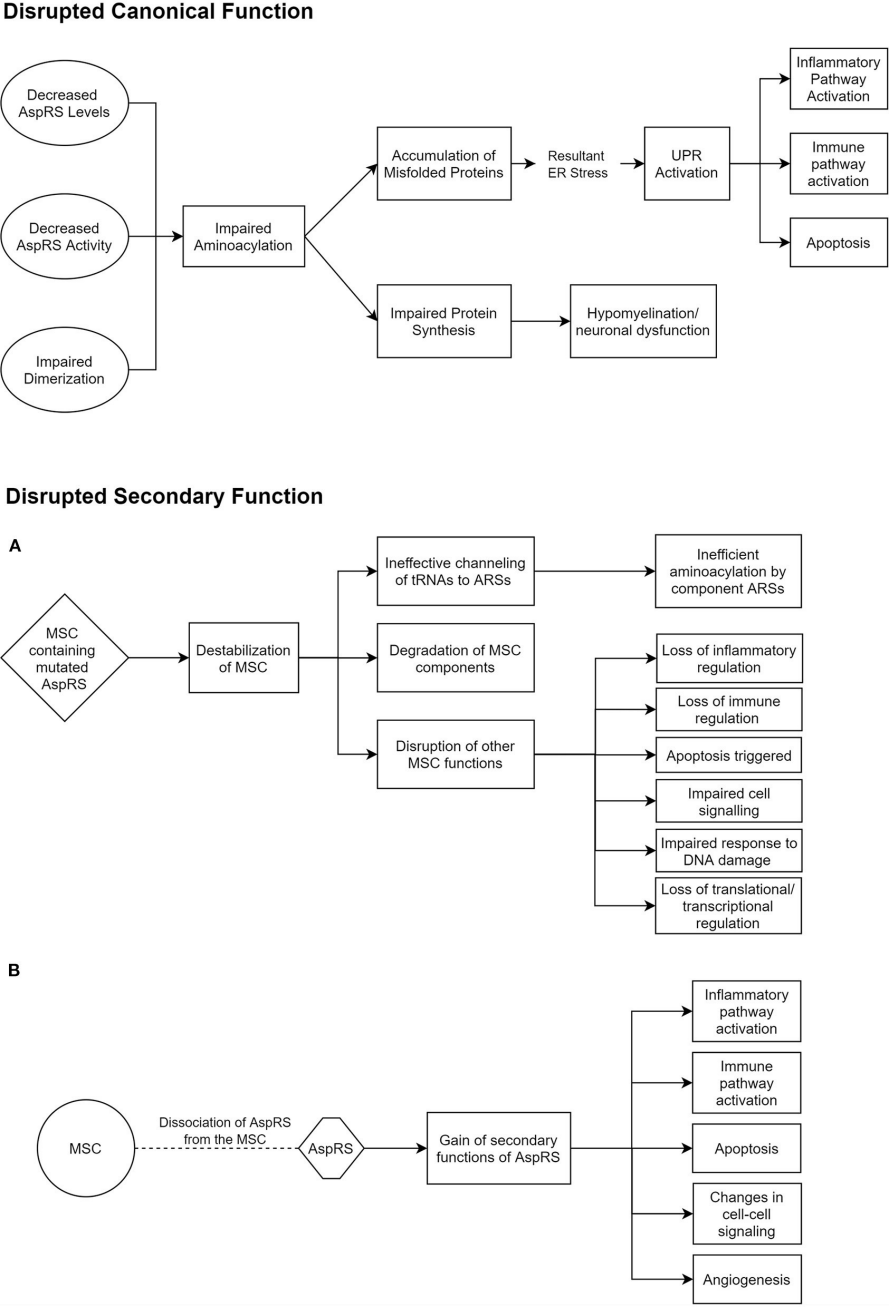
Potential pathomechanisms underlying HBSL. These hypothesized modes of dysfunction in HBSL are based on current knowledge of DARS1, the MSC, and secondary functions of ARSs (Lee et al., 2004; Guo et al., 2010; Pang et al., 2014; Frohlich et al., 2017). **(A,B)** Offer two alternative explanations for the disruption of secondary AspRS function. AspRS, Aspartyl-tRNA Synthetase; ER, Endoplasmic Reticulum; UPR, Unfolded Protein Response; MSC, Multi-tRNA Synthetase Complex; ARS, Aminoacyl-tRNA Synthetase.

ARSs are primarily responsible for the accurate pairing of tRNAs to their respective amino acids, in a two-step aminoacylation reaction (Wallen and Antonellis, 2013). This is often referred to as charging. Some ARSs have additional editing capabilities to identify and correct erroneous charging (Beuning and Musier-Forsyth, 2000). However, such a capability has not been reported for AspRS or mt-AspRS. All studies investigating the mechanism of disease in HBSL and LBSL have so far been mainly targeted toward their canonical function of aminoacylation. There is a growing body of evidence, though, that the secondary ARS functions may also play a role in ARS pathologies (Guo and Schimmel, 2013). One study found missense mutations in *DARS2* regions that are solely conserved in mammals, which could suggest that the evolutionary later-acquired supplementary functions of mt-AspRS may be involved in LBSL pathology (Sauter et al., 2015). These secondary functions include inflammatory and immune regulation by ARSs (Guo and Schimmel, 2013). In addition, cytosolic AspRS is known to interact with other cytosolic ARSs and aminoacyl tRNA synthetase complex interacting multifunctional proteins (AIMPs) to form a multi-synthetase complex (MSC) (Khan et al., 2020). While much of the MSC's function and role is shrouded, it is thought to regulate the secondary functions of the involved ARSs and to promote translation by ferrying tRNAs to ribosomes (Lee et al., 2004). Thus, impaired MSC function could play a role in HBSL (Figure 1).

Compromised aminoacylation may occur as a result of reduced enzyme production, decreased catalytic reaction or impaired dimerization. *In vitro* analysis of seven missense mutations of *DARS2* revealed that protein levels were greatly reduced as a result of the C152F, Q184K, and D560V mutations (van Berge et al., 2013). The D560V mutation additionally caused a decrease of catalytic activity by 83.4% (van Berge et al., 2013) while another study showed a much greater reduction of up to 99.7% (Scheper et al., 2007). The R263Q and L626Q mutations also reduced catalytic activity of mt-AspRS by 99.3 and 97.7%, respectively (van Berge et al., 2013). Reduced mt-AspRS activity was also found in patient-derived lymphoblasts of a compound heterozygous (missense mutations R125H and G338E) LBSL case (Ikazabo et al., 2020), as well as in patients with the highly prevalent splicing defect and other missense mutations (Van Berge et al., 2014). It is notable that not all missense mutations studied resulted in reduced mt-AspRS expression or activity (van Berge et al., 2013). An effect of *DARS1* mutations on AspRS expression levels or activity in HBSL patients has not been reported yet. However, the positions of affected amino acids in and around the catalytic domain strongly suggest that these mutations affect AspRS enzymatic activity (Taft et al., 2013).

In LBSL, impaired transport of mt-AspRS from the cytosol into mitochondria has been suggested as a potential cause of compromised aminoacylation. This disease mechanism was described for the S45G mutation, which is situated in the mitochondrial targeting sequence (MTS) of mt-AspRS (Messmer et al., 2011). The MTS enables nuclear-encoded mitochondrial precursor proteins to be recognized by and transported into mitochondria (Omura, 1998). The S45G mutation impaired the import of precursor mt-AspRS through the mitochondrial membranes, but did not affect targeting of mt-AspRS to the surface of mitochondria (Messmer et al., 2011). A change in mt-AspRS solubility has also been suggested as a potential cause of disease. One study found that the Q184K mutation significantly reduced the solubility of mt-AspRS, while the other four investigated mutations did not substantially affect solubility (Sauter et al., 2015). When combined, two mutations causing only a mild decrease in solubility might be sufficient to trigger LBSL pathology (Sauter et al., 2015). A second study replicated these findings showing that the Q184K mutation significantly decreased the matrix localization of mt-AspRS (Gonzalez-Serrano et al., 2018).

The same study also established that mt-AspRS is localized in the mitochondrial matrix and in the mitochondrial membranes, where it is anchored through electrostatic interactions (Gonzalez-Serrano et al., 2018). This raised the possibility of disrupted ARS localization as a disease mechanism for LBSL (Gonzalez-Serrano et al., 2018). Localization errors could also affect cytosolic ARSs that are part of the MSC. LysRS dissociates from the MSC in response to certain triggers (Ofir-Birin et al., 2013; Gonzalez-Serrano et al., 2018), which subsequently diverts LysRS away from translational sites (Ofir-Birin et al., 2013).

Finally, dimerization is an important aspect to consider in HBSL and LBSL, as both AspRS and mt-AspRS function as homodimers (Bonnefond et al., 2005; Kim et al., 2013). The R58G, T136S, and L626Q mt-AspRS mutations were found to impair dimerization with other mutant mt-AspRS monomers; while the C152F, Q184K, R263Q, and D560V mutations even impacted dimerization with wild-type mt-AspRS subunits (van Berge et al., 2013). None of the reported *DARS1* mutations is located in the dimerization domain of AspRS. However, it has previously been demonstrated in yeast that a change in the highly conserved amino acid P273 in the dimerization domain of AspRS leads to a reduction in catalytic activity of mutant homodimers (Eriani et al., 1993).

Due to the almost identical MRI pattern and similar clinical symptoms, it has been hypothesized that the two conditions could share a common, underlying disease mechanism. Both conditions particularly affect the nervous system, despite ARSs being ubiquitously expressed enzymes. As previously mentioned, in LBSL, the splicing defects seem to be more profound in neurons compared to other cell types (van Berge et al., 2012). The characteristic presentation of LBSL patients without the intron 2 splice site mutation should be further explored. Expression pattern studies of *DARS1* have revealed that neurons have a higher AspRS expression level compared to other cell types in the human brain (Frohlich et al., 2018). On a regional level, the highest AspRS expression was observed in neurons of the cerebellum, particularly in the Purkinje cell layer (Frohlich et al., 2018). To a lesser extent, the motor cortex (predominantly layers II-VI) and hippocampus (CA1, CA2, and CA3 layers) also displayed considerable *DARS1* expression levels. This specific expression pattern may explain why these areas are particularly affected in HBSL (Frohlich et al., 2018). Interestingly, the white matter of the cerebellum and brainstem showed only limited *DARS1* expression (Frohlich et al., 2018). This is in contrast to findings of *DARS2* expression pattern analysis, which was found to be comparable across different tissues (Scheper et al., 2007). However, levels of mitochondrial tRNA levels were found to be highest in the central nervous system (CNS) (Dittmar et al., 2006; Scheper et al., 2007).

## Mouse Models of Disease

Mouse models are useful tools to explore possible disease mechanisms and are a prerequisite for trial of potential therapies. *In vitro* studies have also contributed valuable insight to our current understanding of the diseases, supplementing experiments in mice. Several attempts have been made to model HBSL and LBSL in mice which have provided important mechanistic insights.

The first attempt at an LBSL mouse model found that complete knockout of *Dars2* is lethal; these mice died around embryonic day eight, coinciding with organogenesis (Dogan et al., 2012). Subsequent efforts were directed toward conditional knockout (cKO) of *Dars2* in specific tissues. The first attempt at modeling HBSL in mice encountered the same barrier, as the complete knockout was embryologically lethal due to developmental arrest (Frohlich et al., 2017). This indicates that complete functional null mutations are not tolerated, and the resulting embryonic lethality may explain the rather low numbers of HBSL and LBSL cases diagnosed to date.

Additionally, in both HBSL and LBSL mouse models, the heterozygous null state produced largely phenotypically normal mice (Dogan et al., 2014; Frohlich et al., 2017). This is consistent with the recessive genetic trait of inheritance, that is further supported by the finding that unaffected siblings and parents of patients, who possess one wild-type copy of the *DARS1* or *DARS2* gene, are healthy. Altogether, these findings provide experimental evidence that some residual activity of both these ARSs is necessary for life, and that one unaffected copy of the gene is sufficient to avoid disease.

Two neuronal *Dars2* cKO models have been created utilizing the Cre/LoxP system (Aradjanski et al., 2017; Nemeth et al., 2020). In both models, mice were crossed to CamKIIα Cre-driver lines, expressing Cre recombinase under control of the neuronal CamKIIα promotor. Peak CamKIIα-Cre expression, and hence recombination and *Dars2* knockout, is reached at around postnatal week 4 (Aradjanski et al., 2017). Neuronal *Dars2* cKO mice displayed severe morphological and behavioral changes starting around week 24 and worsening until 28 weeks of age (Aradjanski et al., 2017). These morphological changes included severe brain atrophy concentrated around the forebrain cortex and hippocampal area (Aradjanski et al., 2017; Nemeth et al., 2020) and enlargements of the third and lateral ventricles, with a notable sparing of the cerebellum (Nemeth et al., 2020). Additionally, thinning of the corpus callosum was also noted (Aradjanski et al., 2017; Nemeth et al., 2020). Microscopically, this is accompanied by neuronal cell death, as demonstrated by the presence of pyknotic cells and vacuoles in the affected regions at 28 weeks of age (Aradjanski et al., 2017). In one study, these changes occurred on the background of limited weight gain compared to wild-type mice, and a shorter lifespan. These mice also displayed self-injuries around 28 weeks of age, coinciding with the most severe morphological changes, in addition to motor abnormalities such as tremor and ataxia (Aradjanski et al., 2017). Conversely, in the other study, neuronal *Dars2* cKO mice were of higher body mass than controls until 28 weeks of age, after which they were comparable to controls. In addition, neuronal *Dars2* cKO mice were more active compared to controls, particularly in their explorative behavior (Nemeth et al., 2020). Both models displayed an increase in mitochondria number, however, the mitochondrial ultrastructure appeared normal in one model (Nemeth et al., 2020), while there was a loss of normal cristae in the other model indicating mitochondrial dysfunction (Aradjanski et al., 2017). It remains unclear why the two studies, despite using the same neuronal *Dars2* cKO model, resulted in such different outcomes.

A third neuronal LBSL mouse model specifically targeting Purkinje cells in the cerebellum was recently reported (Rumyantseva et al., 2020). In this model, Cre recombinase was expressed under control of the Purkinje cell protein 2 promotor. Maximal recombination of *Dars2* took place at postnatal weeks 2 to 3 (Rumyantseva et al., 2020). Significant Purkinje cell death was noted at 15 weeks, accompanied by motor dysfunction such as unsteady gait and lack of balance. Morphologically, no cerebellar atrophy was noted (Rumyantseva et al., 2020). Histological analysis revealed a loss of Purkinje cell layer organization at this time. Preceding these changes, mitochondrial respiratory chain (MRC) dysfunction was noted at 6 weeks of age (Rumyantseva et al., 2020). As the cerebellum is the CNS region with the highest AspRS expression in humans and mice, a similar approach to model HBSL in mice should be considered.

It is remarkable that in LBSL patients, mitochondrial dysfunction including altered MRC activity, are not a common feature and have only been reported in one patient to date (Orcesi et al., 2011). Increase in mitochondrial size in muscle cells has also been reported in another patient (Linnankivi et al., 2004). Interestingly, one *in vitro* study revealed a strong reduction in the number of mitochondrial DNA encoded MRC complex proteins in fibroblasts of a compound heterozygous LBSL patient, together with reduced cellular oxygen consumption and increased mitochondrial fragmentation (Lin et al., 2019).

*Dars2* cKO was also performed in the myelinating cells of the central and peripheral nervous system (PNS) at the age of 4 weeks, using tamoxifen inducible Cre expression under control of the proteolipid 1 (*Plp1*) promoter. Similar to the neuronal *Dars2* cKO model produced by the same group, strong MRC deficiencies were observed in these mice, yet no other sequelae involving demyelination, apoptosis or inflammation followed (Aradjanski et al., 2017). Morphologically and behaviorally, these mice were normal too. It was suggested that myelinating cells possess a different metabolic profile compared to neurons, which enables them to produce sufficient energy through glycolysis when mitochondrial respiration is impaired. Mitochondria in mature oligodendrocytes may be more important for myelin maintenance through fatty acid oxidation and lipid synthesis rather than ATP production (Rinholm et al., 2016; Aradjanski et al., 2017). Since *Dars2* cKO only affects translation of mitochondrial DNA transcripts, and hence MRC subunit function and ATP production, myelination remained largely unaffected. Expression levels of cytosolic AspRS are much lower in oligodendrocytes compared to neurons (Frohlich et al., 2018). It has been hypothesized that the low expression levels could make these cells particularly susceptible to disturbances in protein synthesis. However, given the growing evidence of the influence of neurons on oligodendrocyte differentiation and myelination (Gibson et al., 2014; Mitew et al., 2014; Ortiz et al., 2019), it is also plausible for the primary pathology to lie in neuronal cell bodies and axonal processes. This is supported by the predominant involvement of long tracts in LBSL and HBSL (van Berge et al., 2012).

*Dars2* cKO was also achieved in cardiac and skeletal muscles using the muscle creatine kinase (Ckmm) Cre-driver line. Maximal recombination of the *Dars2* gene occurred at 2 weeks of age in cardiac and skeletal muscle (SkM) cells (Dogan et al., 2014). While no cardiac defects have been reported in LBSL patients, muscle tissue has a similar energy demand and therefore findings from this study could be translationally relevant for LBSL. All mice developed hypertrophic cardiomyopathy by 6 weeks of age, quickly followed by death (Dogan et al., 2014). Microscopically, the response in each muscle tissue differed. Cardiac muscle fibers were hypertrophic and disorganized progressively, while atrophy and preserved fiber organization was observed in SkM fibers. Both the cardiac and skeletal myocytes experienced substantial MRC deficiency; however, mitochondrial biogenesis, cell stress pathway activation and suppressed autophagy was only observed in cardiomyocytes (Dogan et al., 2014). It is postulated that this absence, or rather significant delay in onset of regulatory changes is owed to the skeletal myocytes' low mitochondrial transcript turnover rate and the cells' much greater capacity to regenerate (Dogan et al., 2014).

The morphological, behavioral, and microscopic changes observed in these studies have been consistently preceded by molecular changes—mainly the activation of several stress pathways accompanied by immune and inflammatory responses (Dogan et al., 2014; Aradjanski et al., 2017; Nemeth et al., 2020). In the neuronal *Dars2* cKO model, these changes occur as early as 16 weeks of age and particularly impact immune pathways (Nemeth et al., 2020). The most notable change was the upregulation of Cystatin F (CST7). CST7 is a lysosomal protease inhibitor that is known to repress lysosomal proteases to prevent tissue damage (Kos et al., 2018). In the CNS, *Cst7* has been shown to be expressed, and then released from activated microglia (Nemeth et al., 2020). This typically occurs in areas of active demyelination and simultaneous remyelination (Ma et al., 2011). Interestingly, when the remyelinating potential is lost (such as in chronic demyelination), CST7 levels fall (Ma et al., 2011). Therefore, the reported 200-fold increase of CST7 levels in neuronal *Dars2* cKO mice (Nemeth et al., 2020) may indicate the enduring potential for remyelination. However, despite the CST7 increase, the neuronal *Dars2* cKO mice continued to deteriorate. Another role of CST7 is the regulation of cathepsin C (CTSC)—a known lysosomal cysteine protease, which was also upregulated in neuronal *Dars2* cKO mice (Nemeth et al., 2020). CTSC is involved in the activation of several immune cells, including T cells and neutrophils, and its absence has been shown to dampen the immune response (Colbert et al., 2009). One study found the knockout of *Ctsc* “rescued” the demyelinating process in multiple sclerosis (Shimizu et al., 2017). The upregulation of CTSC could potentially contribute to the progression of LBSL.

These immune changes coincided with signs of inflammation. In histological brain sections of neuronal *Dars2* cKO mice, a significant increase in the number of activated microglia expressing the inflammatory marker CD68 was observed (Nemeth et al., 2020). Although the role of CD68 in inflammation is unclear, it is known to be upregulated in macrophage-mediated inflammatory processes and is hence a reliable indicator for microglia activation (Chistiakov et al., 2017). This finding is further corroborated by the second neuronal *Dars2* cKO study, where increasing microglial activation was observed in the cortex until 20 weeks of age, when neuronal cells began to undergo apoptosis (Aradjanski et al., 2017). These inflammatory changes resulted in a near complete disruption of normal cellular structures in the hippocampal area close to the time of death (Aradjanski et al., 2017), and even contributed to cell-death in CamKIIα negative neurons (Nemeth et al., 2020). In the Purkinje cell *Dars2* cKO mouse model, the observed microglial activation and associated neuroinflammatory changes have been attributed to degradation of Purkinje cell dendrites and their complex network (Rumyantseva et al., 2020).

In addition, molecular changes indicative of mitochondrial dysfunction and disrupted proteostasis have been observed. Following conditional knockout of *Dars2* in cardiac and SkM, the activation of the mitochondrial unfolded protein response (UPR^mt^) in cardiomyocytes was evident by 6 weeks of age (Dogan et al., 2014). Stress pathway activators such as activation transcription factor 5 (ATF5) and C/EBP homologous protein (CHOP) were found to be upregulated at this time point (Dogan et al., 2014). These molecules stimulate the UPR^mt^, which is responsible for the degradation of aberrant proteins in order to maintain cell homeostasis (Haynes and Ron, 2010). The activation of the UPR^mt^ was the first notable change in mutant cells and increased ATF5 and CHOP levels along with proteases involved in the UPR^mt^ were identified before any detectable MRC deficits (Dogan et al., 2014). In the neuronal *Dars2* cKO model, molecular changes suggestive of UPR^mt^ activation were also observed at 20 weeks of age coinciding with severe MRC defects (Aradjanski et al., 2017). Accordingly, the activation of the unfolded protein response (UPR) as a possible disease mechanism for HBSL was suggested (Frohlich et al., 2017).

A 250-fold increase of fibroblast growth factor 21 (FGF21) within the first 2 weeks of life in the heart of Ckmm *Dars2* cKO mice is further evidence of mitochondrial dysfunction preceding detectable MRC deficiency (Dogan et al., 2014). FGF21 has been shown to be elevated in mitochondrial diseases, particularly those affecting the muscles, and has even been suggested as a biomarker for mitochondrial disorders for its high sensitivity and specificity (Suomalainen et al., 2011). Notably, most of the FGF21 increase resulted from cardiomyocytes, where *Fgf21* expression levels are usually low (Dogan et al., 2014). FGF21 is predominantly known for its role in modulating metabolic pathways (Potthoff et al., 2009). This is also evident in Ckmm *Dars2* cKO mice through altered nutrient usage (Dogan et al., 2014). Additionally, FGF21 ensures the stability of the transcription factor Peroxisome proliferator-activated receptor-Gamma Coactivator 1-alpha (PGC-1α) (Fisher et al., 2012). PGC-1α is a key controller of mitochondrial biogenesis (Ventura-Clapier et al., 2008)—an adaptive response to increased ATP demand.

In addition to the UPR, the integrated stress response (ISR) pathway was also activated in neuronal *Dars2* cKO mice. This was evident through the upregulation of *Atf4*, a key component of the ISR, at 16 weeks of age (Nemeth et al., 2020). ISR activation also occurred prior to any observable neuronal death. It is remarkable that only increased mitochondrial biogenesis, without any mitochondrial dysfunction, was noted in this model. In the Purkinje cell *Dars2* cKO mouse model, ISR activation was noted at 8 weeks of age, as evidenced by the initiation of the one-carbon metabolic pathway. This pathway has been linked to the activation of the ISR through *Atf4* upregulation (Rumyantseva et al., 2020). The ISR is a cell stress pathway intended to maintain homeostasis and is activated upon dysregulation of proteostasis, oxidative damage, loss of nutrients, and other similar events (Costa-Mattioli and Walter, 2020). When the challenge becomes unmanageable, apoptosis is triggered. Intriguingly, prolonged activation of this usually protective pathway can in itself become cytotoxic, which has been shown to be the case in several neurodegenerative disorders (Rabouw et al., 2019).

**Figure 2:**
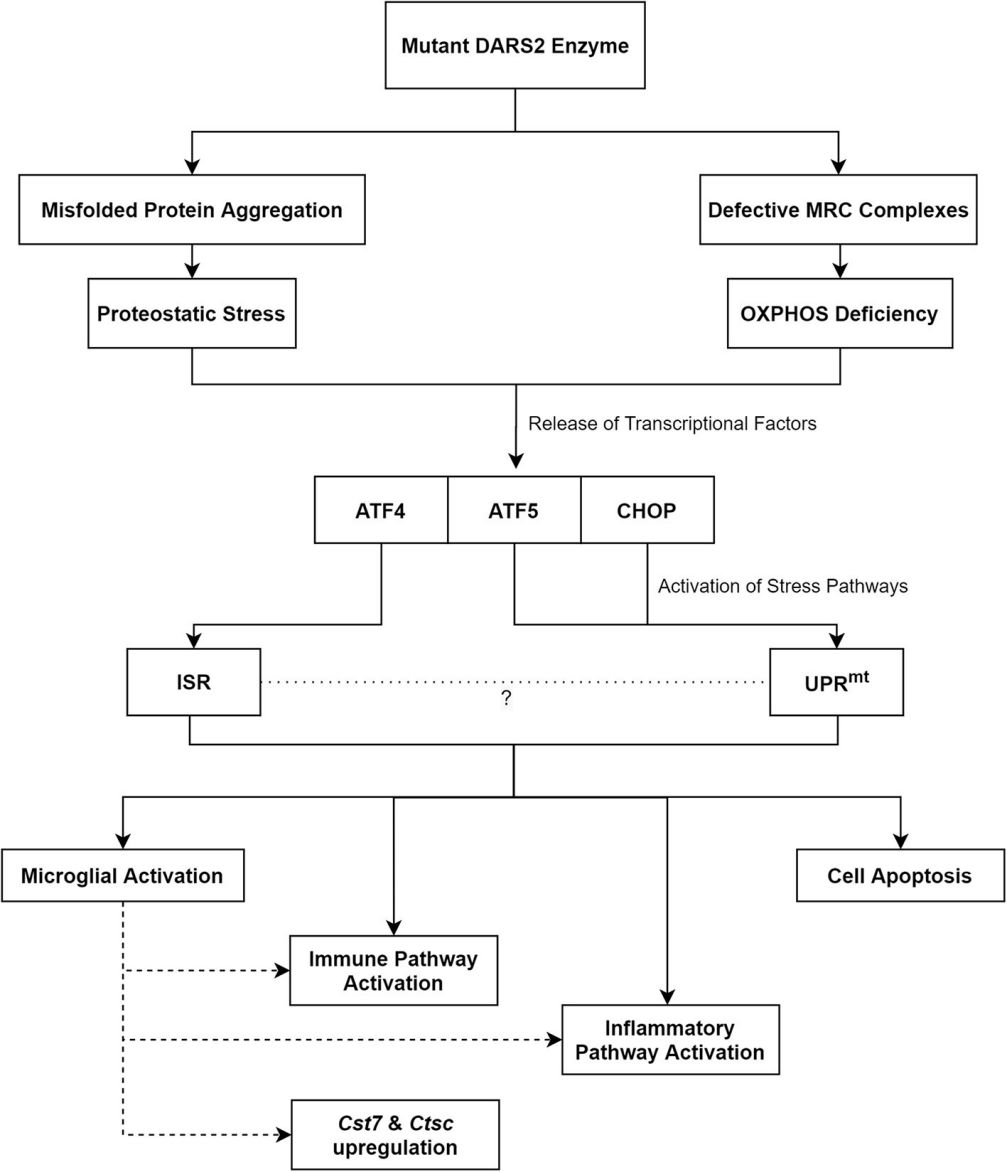
The proposed pathomechanisms of LBSL based on findings from the neuronal *Dars2* cKO mouse models. MRC, Mitochondrial Respiratory Chain; OXPHOS, Oxidative Phosphorylation; ATF, Activation Transcription Factor; CHOP, C/EBP Homologous Protein; ISR, Integrated Stress Response; UPR^mt^, Mitochondrial Unfolded Protein Response; Cst7, Cystatin F; Ctsc, Cathepsin C.

Taken together, an imbalance in protein homeostasis, which is evident through a rise in ubiquitinated proteins, appears to be the initial trigger for the disease (Dogan et al., 2014). Subsequently, this leads to the activation of stress pathways, which through prolonged activation likely contributes to neuronal cell-death and neurodegeneration (Figure 2).

The neuronal *Dars2* knockout mouse model generated by Aradjanski et al. was able to capture some of the LBSL symptomatology seen in patients, such as ataxia and tremor (Aradjanski et al., 2017). Neuronal *Dars2* cKO mice also displayed worsening kyphosis with age. Signs of microglial activation and cell death corroborated the histological findings in LBSL patients (Yamashita et al., 2013). Still, gross pathological changes were mainly limited to cortical and hippocampal areas, while LBSL patients displayed WM changes in the cerebellum, brainstem and spinal cord in addition to cortical changes. This discrepancy can be explained by the predominant localization of CamKIIα positive neurons within the hippocampus and cortex, while the midbrain, medulla and cerebellum lack CamKIIα positive neurons (with the exception of Purkinje cells) (Wang et al., 2013). The use of a more appropriate neuronal Cre-driver to ablate *Dars2* in all neurons of the CNS might result in a more accurate LBSL model. Surprisingly, despite using the same CamKIIα Cre-driver, Nemeth et al. demonstrated increased activity of neuronal *Dars2* cKO mice without any overt motor deficits (Nemeth et al., 2020). It is also important to bear in mind that regulation of mRNA translation in the human brain likely varies from the mouse brain.

For HBSL, our lab is currently working on a *Dars1* cKO mouse model. In addition, we are attempting to model HBSL by introducing patient specific point mutations into the mouse *Dars1* gene. The first mutation we introduced was the *Dars1*^*D*367*Y*^ point mutation found in the HBSL index patient (see Froehlich et al., in this issue). While homozygous *Dars1*^*D*367*Y*/*D*367*Y*^ mice failed to model HBSL, mice compound heterozygous for the *Dars1*^*D*367*Y*^ and the *Dars1*^*null*^ mutation captured some aspects of the disease.

In summary, attempts at modeling HBSL and LBSL have contributed significantly to our understanding of both conditions. Nevertheless, these studies have their limitations. In all studies, *Dars2* was rendered functionally inactive postnatally. Since CNS development is accelerated in mice compared to humans, these knockouts occur relatively late during development. In contrast, *DARS2* mutations are evident from birth in LBSL patients. These mutations greatly reduce, but do not completely abolish mt-AspRS function, throughout the developmental period.

In mice, complete knockout of *Dars1* or *Dars2* were both embryonically lethal. This indicates a lack of functional redundancy between the two enzymes and puts to rest questions on possible compensatory mechanisms between the two. However, other findings from these studies continue to offer support for a possible shared disease mechanism between LBSL and HBSL. Firstly, the finding of inflammatory processes and immune responses likely underlying neuronal dysfunction and cell-death in LBSL is reminiscent of the study by Wolf et al., who postulated that HBSL might possess a neuroinflammatory component based on clinical findings and the responsiveness of HBSL patients to steroids (Wolf et al., 2015). This should be addressed when studying the underlying disease mechanism in future HBSL mouse models.

Secondly, studies in LBSL models suggest the primary pathology to lie in neuronal or axonal dysfunction rather than in myelin-producing cells or the myelin itself. These findings are supported by the absence of noticeable phenotypic changes in mice following *Dars2* cKO in myelinating cells. Moreover, one study reported findings of axonal degeneration of myelinated fibers in histopathological analysis of tissues from LBSL patients. This was accompanied by detection of myelin ovoids in the peripheral sural nerve, and the absence of segmental demyelination, hence the pattern of peripheral neuropathy in these patients was suggested to be mainly axonal (Yamashita et al., 2013). Myelin ovoids are often associated with Wallerian degeneration—a pathological process where demyelination occurs secondary to axonal pathology. Myelin ovoids result from the clearance of myelin by Schwann cells (Tricaud and Park, 2017). Segmental demyelination indicates myelin degeneration with sparing of nerve fibers, yet this was not a prominent finding in the histological analysis (Yamashita et al., 2013). Accordingly, AspRS was found to be predominantly expressed by neurons, with lower expression levels in oligodendrocytes and astrocytes. In mice, the subcellular localization of AspRS was not restricted to the cell body of neurons but also in the axons and synapses (Frohlich et al., 2017). Taken together, there is a strong rationale for a primary neuronal or axonal dysfunction underlying LBSL and HBSL pathology.

## Therapies and Management

Management of HBSL and LBSL are currently solely supportive and symptomatic. While these methods can improve the quality of life, the benefits are neither sustained nor equally effective for all patients. Hence, there is a need for the development of novel treatment strategies for patients affected by these conditions. Some medications have been shown to be effective in patients and when tested *in vitro* for LBSL (Synofzik et al., 2011; Van Berge et al., 2014). As previously stated, for the investigation and trial of new curative treatment options accurate animal models are required to conduct pre-clinical studies. In the HBSL mouse model study, phenotypically normal heterozygous null mice possessed just 20% of AspRS protein levels compared to control mice (Frohlich et al., 2017). As such, reinstating the enzyme to this “threshold” level might be sufficient to ameliorate symptoms or prevent disease.

For the symptomatic management of LBSL, the use of allied health specialties such as speech, nutrition and physiotherapy has been reported, to support patient disability (Navarro Vázquez et al., 2016; Werner et al., 2018). In addition, medications for pain and spasticity are also used for symptom management (Werner et al., 2018). Antispastic medications have been shown to improve symptoms gradually in adult-onset LBSL patients (Petzold et al., 2006). At the same time, genetic counseling is often provided to affected families. In HBSL, management is similarly symptom-based, with chemodenervation additionally suggested to manage spasticity (Ulrick and Vanderver, 2017).

In both conditions, steroid medications have been shown to reduce the severity of symptoms experienced by patients. Particularly in HBSL, 5 out of 16 patients reported in the literature showed improvements following administration of steroids, three of whom were infantile-onset patients (Taft et al., 2013; Wolf et al., 2015). One early-onset patient was described to gain muscle strength and postural tone, although these improvements were only modest (Wolf et al., 2015). In late-onset patients, the effect of steroids was inconsistent—with one patient regaining his ability to walk independently following a single course of methylprednisolone, while another patient repeatedly experienced relapse of symptoms over a few months post-steroid treatment (Wolf et al., 2015). In LBSL, to our knowledge, the use of steroids has only been described in one patient who displayed spastic bladder symptoms, which improved following the use of glucocorticoids, although other symptoms persisted (Cheng et al., 2013). Following the uncovering of activated inflammatory and immune pathways in LBSL mouse models, the use of steroids could be beneficial for symptom management in LBSL patients in the future. Concomitantly, variable suppression of symptoms through the use of steroids in HBSL hints at inflammatory and immune activation as a component of the disease process. Further investigation into the cause of the activation of inflammatory and immune pathways—such as the UPR and ISR—will help develop more effective treatment options. An ISR inhibitor (ISRIB) is of interest, as it has delivered promising results in inhibiting the harmful effects of ISR overactivation without producing excessive side effects from inhibition of healthy, basal ISR protection (Rabouw et al., 2019).

Lately, some drugs have gained special attention for the treatment of LBSL. Firstly, the carbonic anhydrase inhibitor acetazolamide was reported to improve ataxia in one patient (Synofzik et al., 2011). This medication, however, was used in a patient with a highly atypical presentation, where ataxia was episodic and triggered by exercise. In such a patient, 125 mg bi-daily acetazolamide was shown to decrease the mean number of ataxic attacks per day by about 77%. Acetazolamide is a standard medication used in episodic ataxia syndromes, for which this patient did not harbor mutations, and is thought to exert its effect in the CNS by reducing lactate and pyruvate levels (Griggs et al., 1978). Notably, this patient displayed lactate elevation on MRS. It remains to be seen if this medication will produce similar results in more typical LBSL patients. Another drug, cantharidin, has been demonstrated to improve the efficiency of splicing at the *DARS2* intron 2 splice site *in vitro* (Van Berge et al., 2014). There was a 5.9% increase in the inclusion of exon 3 in mRNA transcripts of *DARS2* following cantharidin application. While cantharidin is toxic and unsuitable for clinical use, the use of other protein phosphatase 1 and 2A inhibitors could be explored for therapeutic use. Alternatively, antisense oligonucleotides have also been shown to increase the efficiency of splicing at this site (van Berge et al., 2012), although their use is limited by their inability to cross the blood-brain barrier (Rinaldi and Wood, 2018). As the intron 2 splice site mutation is the most prevalent mutation reported in LBSL patients, this site is a valuable target for the treatment of LBSL (Van Berge et al., 2014). For HBSL, it has been suggested that boosting AspRS activity through nutraceutical L-ornithine-L-aspartate (LOLA) supplementation might have therapeutic effects (Das et al., 2020).

Finally, gene therapy is often considered for inherited conditions. This is a particularly viable option for HBSL and LBSL, as they are both monogenic disorders and therefore highly amenable to such a treatment approach. With the recent advances in creating animal models for HBSL and LBSL, attempts at establishing such a treatment modality will follow and hopefully provide a cure for these devastating conditions.

## Author Contributions

AM and DF led the project and the manuscript production. MK and GH contributed to the manuscript preparation. All authors read and approved the final manuscript.

## Conflict of Interest

MK is an employee of Boehringer Ingelheim Pharma GmbH & Co. KG. The remaining authors declare that the research was conducted in the absence of any commercial or financial relationships that could be construed as a potential conflict of interest.
